# Health Measurement and Health Inequality Over the Life Course: A Comparison of Self-rated Health, SF-12, and Grip Strength

**DOI:** 10.1007/s13524-019-00761-x

**Published:** 2019-03-05

**Authors:** Liliya Leopold

**Affiliations:** 1 Department of Sociology, University of Amsterdam, Amsterdam, the Netherlands; 2 Department of Sociology, University of Bamberg, Bamberg, Germany

**Keywords:** Self-rated health, Grip strength, Health inequality, Life course

## Abstract

The cumulative (dis)advantage hypothesis predicts education differences in health to increase with age. All previous tests of this hypothesis were based on self-reported health measures. Recent research has suggested that self-reported health measures may not adequately capture differences in key analytical constructs, including education, age, cohort, and gender. In this study, I tested the cumulative (dis)advantage hypothesis using a self-reported subjective measure (self-rated health), a self-reported semi-objective measure (PCS based on SF-12), and an objective measure (grip strength) of general physical health. Hierarchical linear models applied to five waves of panel data (SOEP, 2006–2014, *N* = 3,635 individuals aged 25 to 83, comprising *N* = 9,869 person-years) showed large differences between health measures. Among men, education differences in both self-reported measures of health widened substantially with age, consistent with the cumulative (dis)advantage hypothesis. For grip strength, education differences were small and changed little with age, inconsistent with the hypothesis. Among women, education differences in both self-reported measures of health remained stable over the life course, but they widened substantially when measured by grip strength. I conclude that evidence on the cumulative (dis)advantage hypothesis is sensitive to the choice of a health measure.

**Electronic supplementary material:**

The online version of this article (10.1007/s13524-019-00761-x) contains supplementary material, which is available to authorized users.

## Introduction

A large literature has examined social inequality in trajectories of physical health. Several studies have shown that health gaps between education groups grow with age (Chen et al. 2010; Kim 2008; Leopold 2016; Mirowsky and Ross 2008; Willson et al. 2007). This evidence is consistent with the *cumulative (dis)advantage hypothesis*, which states that education differences in determinants of health—such as living and working conditions, exposure to stress, social support, and health behaviors—translate into increasing physical health differences between higher- and lower-educated people over the life course (Ross and Wu 1996). This hypothesis has become the dominant theoretical framework in life course research on health inequality (Dannefer 2003; DiPrete and Eirich 2006; Ferraro et al. 2009).

An important shortcoming of this line of research is that empirical tests of the cumulative (dis)advantage hypothesis are limited to self-reported measures of physical health. Most studies have used self-rated health, commonly measured on a 5-point scale, as the only or as the primary health outcome (Brown et al. 2016; Chen et al. 2010; Goesling 2007; Leopold 2016; Leopold and Leopold 2018; Lynch 2003; Sacker et al. 2011; Torres et al. 2016; Willson et al. 2007). Although the benefits of self-rated health are widely recognized, recent studies have raised doubts about the validity of this measure in studies of health inequality over the life course (Frick and Ziebarth 2013; Juerges 2010).

Four limitations of self-rated health have been highlighted. First, this measure may not adequately capture differences between socioeconomic groups (Dowd and Zajacova 2007; Huisman et al. 2007; Singh-Manoux et al. 2007). Health differences between higher- and lower-educated people, as measured by biomarkers, were found to be underestimated by a measure of self-rated health (Dowd and Zajacova 2010). Second, self-rated health may not adequately capture gender differences. For example, men and women were shown to attach different weight to different aspects of physical health when reporting on self-rated health (Peersman et al. 2012). Third, self-rated health may not adequately capture change over the life course. In a recent study, the predictive power of poor self-rated health for mortality—one of the most common arguments to establish its validity as a health measure—was found to decline with age (Zajacova and Woo 2016). Fourth, self-rated health may not adequately capture change across cohorts. Recent research has indicated that self-evaluations of health became more valid across cohorts (Schnittker and Bacak 2014). Taken together, these four problems raise doubts about the robustness of previous findings on education differences in physical health trajectories as measured by respondents’ self-assessments.

Although the limitations of self-reported health measures are increasingly understood, no study has examined the extent to which these measures affect the main conclusions that have emerged from life course research on health inequality. The present study aims to fill this gap of knowledge. Specifically, I examined whether and to what extent age trajectories of physical health, and education differences therein, depended on the choice of a health measure. I compared three outcome measures: (1) self-rated health as a reference measure for subjective evaluations of general health; (2) the Physical Component Scale (a physical health index based on the SF-12 instrument) as a self-reported quasi-objective health measure; and (3) grip strength as an objective measure of muscle strength and general physical health assessed by a dynamometer.

To compare these measures, I used data from the German Socioeconomic Panel Study (SOEP), in which all three measures are available biannually from 2006 until 2014. I selected a sample of 3,635 individuals and 9,869 observations (person-years) in which data on all three measures were available. To analyze changes in education differences in physical health, I used hierarchical linear models and age-vector graphs mapping trajectories of each health measure across an age range between 25 and 83 for West German men and women born between 1931 and 1981.

## Background

### Education and Health Over the Life Course

The question of whether education differences in health increase or decrease with age has been examined for more than three decades. Initial research was motivated by two competing hypotheses: the cumulative (dis)advantage hypothesis and the age-as-leveler hypothesis. The cumulative (dis)advantage hypothesis states that education structures the distribution of determinants of health, such as living and working conditions, exposure to stress, social support, and health behaviors, which translates into increasing physical health differences between higher- and lower-educated people over the life course (Ross and Wu 1996). The *age-as-leveler hypothesis* states that education differences in health increase only until early old age but decline thereafter (House et al. 1994). This hypothesis stresses the importance of social policy and selection effects. Social policy arguments concentrate on institutional interventions, such as Medicare (providing more equal access to health care) or Social Security (alleviating economic inequality among older adults) (Dannefer 2003). The selection argument attributes the decline of education health differences in older age to selective mortality and selective participation in surveys (Kitagawa and Hauser 1973; Wilkinson 1986).

Empirical tests of these hypotheses have focused on aggregate-level patterns rather than directly testing the individual-level mechanisms proposed by these hypotheses. Education gaps that widened with age have been regarded as support for the cumulative (dis)advantage hypothesis, whereas education gaps that narrowed with age have been regarded as support for the age-as-leveler hypothesis.

Pioneering research on education inequality in health trajectories has produced mixed findings and fueled an intense debate. In the course of this debate, theoretical views and empirical tools have been refined. Methodologically, the most important conclusion is that cross-sectional or short-term longitudinal data are not well suited to examine health trajectories of education groups. Longitudinal data are necessary to account for selection effects and to disentangle age effects from cohort effects because education differences in health trajectories were found to change across cohorts (Lynch 2003; Noymer 2001).

The finding of narrowing health gaps between education groups in older age is no longer considered to contradict the cumulative (dis)advantage hypothesis, as long as this trend results from selective mortality. Instead, higher rates of mortality among the lower-educated are seen as an outcome of processes described by the cumulative (dis)advantage perspective (Dupre 2007; Ferraro et al. 2009; Lynch 2003; Rohwer 2016; Willson et al. 2007). Thus, processes of cumulative (dis)advantage may suppress age-related increases in health inequality.

Current empirical tests of the cumulative (dis)advantage hypothesis assess whether education differences increase before later-life stages in which differential mortality may narrow the gaps. Most of the evidence comes from the United States and is consistent with aggregate-level health trajectories implied by the cumulative (dis)advantage hypothesis. Moreover, U.S. studies have found that this pattern is more pronounced among women and has intensified across cohorts (Brown et al. 2016; Liu and Hummer 2008; Ross and Mirowsky 2010). Evidence from the West German context of the present study, instead, points to a stronger age and cohort increase of education differences in self-rated health among men. Among women, education gaps in health do not widen with age, and this does not change across cohorts (Leopold and Leopold 2018).

Taken together, the advances from previous studies suggest that research on education differences in physical health trajectories should (1) use longitudinal data to account for selection effects and to separate age effects from cohort effects, and (2) examine gender differences given that conclusions about education differences may differ between men and women. In contrast to variation in estimation methods, variation across cohorts, and variation by gender, however, no systematic study has explored how variation in health measures affects conclusions about education differences in trajectories of physical health.

### Does the Health Measure Matter?

Most studies have examined change in self-rated health, a measure of general physical health (Brown et al. 2016; Chen et al. 2010; Goesling 2007; Leopold 2016; Leopold and Leopold 2018; Lynch 2003; Mirowsky and Ross 2008; Sacker et al. 2005; Torres et al. 2016; van Kippersluis et al. 2009, 2010; Willson et al. 2007). The main reasons for the dominant role of this measure in the literature are (1) data availability, because self-assessments of health are often included in the core questionnaires of long-running panel studies; (2) validity, because self-rated health correlates with current and future health problems and mortality (Idler and Benyamini 1997; Mossey and Shapiro 1982); and (3) life course coverage, because self-rated health captures health differences across the entire lifespan. This contrasts with measures of specific conditions, symptoms, and functional limitations that become prevalent only at advanced ages (Willson et al. 2007).

Despite these benefits, recent research has raised doubts about the measure of self-rated health. The most important concern is a key assumption underlying studies on inequality: namely, that respondents in different social groups assess their overall health status in similar ways and on the basis of similar criteria, independent of their education level, age, cohort, and gender. An increasing number of studies have suggested that this assumption might be unwarranted (Au and Johnston 2014; Burgard and Chen 2014; Zajacova and Woo 2016).

The first problem concerns education differences in the validity of self-rated health. Studies have shown that for lower-educated persons, this measure is less predictive of mortality (Dowd and Zajacova 2007), and its association with physical health problems as assessed by biomarkers is weaker than for higher-educated persons (Dowd and Zajacova 2010). These findings suggest that education differences in physical health may not be adequately captured by a measure of self-rated health (Dowd and Zajacova 2010; d’Uva et al. 2008; Molina 2016).

Second, increasing evidence suggests that the validity of self-rated health depends on age. As people get older, their frame of reference for assessing their health status changes, and individuals tend to overestimate their health despite an increasing number of physical health problems (Krause and Jay 1994; Peersman et al. 2012). Accordingly, the power of poor self-rated health to predict chronic diseases and mortality weakens substantially with age (Helweg-Larsen et al. 2003; Idler and Cartwright 2018; Lindeboom and van Doorslaer 2004; Schnittker 2005; Van Doorslaer and Gerdtham 2003; Zajacova and Woo 2016).

Third, self-ratings of health vary across cohorts. As demonstrated in a recent study, the predictive power of self-rated health for mortality increases among more recently born (Schnittker and Bacak 2014). The explanation proposed by this study is that because of education expansion, improvements in health knowledge, and medical progress, younger cohorts are better informed about their physical health status and thus provide more accurate self-assessments.

Finally, some doubts have been raised regarding the validity of self-rated health as a measure of gender differences in health. This evidence, however, is not consistent. One recent study found no gender differences in terms of criteria that individuals use to report on their self-rated health status Zajacova et al. 2017). Other studies found that women take milder symptoms and health complaints into account, whereas men focus on more severe or even life-threatening conditions as well as on health behaviors (Benyamini et al. 2003; Grol-Prokopczyk et al. 2011; Peersman et al. 2012). These findings indicate that self-rated health measures may not adequately capture gender differences in physical health.

Taken together, the evidence from these studies questions whether self-rated health accurately measures differences in terms of education, age, cohort, and gender. Given that all of these four factors represent key analytical constructs in studies of health inequality over the life course, this raises doubts about the conclusions of previous research. In studies on the cumulative (dis)advantage hypothesis, however, these issues are rarely considered because self-rated health remains the primary outcome measure.

A few studies have complemented or replaced self-rated health with alternative measures, although these measures have also relied on respondents’ self-reports (House et al. 2005; Hu et al. 2016; Kim 2008; Kim and Durden 2007; Leopold 2016; Torres et al. 2016; van Kippersluis et al. 2009). Among the measures used are different indices of self-reported physical impairments or functional limitations. These measures are based on a battery of questions about limitations in daily activities, such as walking several blocks, climbing several flights of stairs, lifting something heavy, or picking up a coin from a table. Studies on the intergroup validity of these measures, however, have suggested that concerns about self-rated health may apply similarly to self-reported measures that are based on specific health problems (Burgard and Chen 2014; Molina 2016; Ziebarth 2010).

A potential solution to these problems is to complement subjective measures by objective measures of health. Although no study has used objective measures in tests of the cumulative (dis)advantage hypothesis, the literature suggests that they may constitute a suitable alternative to self-reported measures of health given that they are not affected by systematic group differences in reporting behavior (Burgard and Chen 2014; Ploubidis and Grungy 2011).

In particular, grip strength (as assessed by a dynamometer) is considered a valid indicator of general physical health status that captures changes in health across adulthood (Peterson et al. 2016). Because of its potential as a low-cost and noninvasive objective measure of overall health in population studies, the properties and validity of grip strength have been studied extensively over the past decade. Meta-studies have shown that similar to self-rated health and other self-reported health measures, grip strength predicts all-cause and cause-specific mortality, and correlates with current and future disability (Bohannon 2001; de Lima et al. 2017; Rijk et al. 2016; Wu et al. 2017). Moreover, grip strength was found to be strongly related to aging, declining from mid-adulthood onward (Steiber 2016; Vianna et al. 2007).

The main factors that underlie the decline of grip strength with age are reductions in muscle strength and skeletal muscle mass (Abe et al. 2014). This decline reflects biological processes that lead to a number of physical conditions, diseases, and causes of death. First, declining muscle strength is caused by a decrease in serum levels of testosterone and adrenal androgens, increased action of inflammatory mediators, and the onset of anabolic resistance (Montalcini et al. 2013; Schlüssel et al. 2008). Changes in these factors are often caused by declines in physical activity as a result of chronic and acute diseases, injuries, and (to a smaller degree) stress and depression (Syddall et al. 2003). Moreover, low grip strength is a strong predictor of falls, physical disability, and frailty, all of which lead to increased mortality risk, especially in old age.

Second, grip strength is an indicator of skeletal muscle mass, which is responsible for the disposal of blood glucose. The amount of skeletal muscle mass indicates the ability to respond to insulin, which in turn is a major predictor of metabolic diseases and mortality from metabolic diseases, such as type 2 diabetes. Moreover, sarcopenia (degenerative loss of skeletal muscle mass) is associated with chronic low-grade inflammation, which occurs in a number of common chronic diseases that lead to premature mortality (Metter et al. 1997; Peterson et al. 2016).

Although grip strength is per definition not affected by reporting heterogeneity, biological differences between men and women have to be taken into account. Men have more muscle mass than women because of higher plasma concentrations of the major anabolic hormones (testosterone, GH, and IGF-1) but also because of more physical activity on the job and during leisure time (Montalcini et al. 2013). Moreover, men (but not women) with higher body weight and higher levels of body fat—common predictors for health impairment—have higher grip strength (Gale et al. 2007). Although gender differences in the predictive validity of grip strength as a general health measure are not fully understood, the most recent and comprehensive meta-study has concluded that there are no substantial gender differences in the relationship between grip strength and all-cause mortality as well as mortality from cardiovascular diseases, stroke, coronary heart disease, and cancer (Wu et al. 2017).

Taken together, the literature on health measures suggests that grip strength may accurately capture group differences arising from the unequal distribution of health-related resources and exposures to health risks over the life course. These benefits render grip strength an interesting alternative to self-rated health or other self-reported health measures to study the core association between education and health, as well as how it changes with age, across cohorts, and for men and women.

## Methods

### Data and Sample

The analysis was based on data from the German Socio-Economic Panel Study (SOEP), a large-sсale study of individuals and households (Wagner et al. 2007). The SOEP started in 1984 and collects data that are representative of the population of Germany who are older than 16 years. Data on self-rated health were collected annually since 1992, data on PCS were collected biannually since 2002, and data on grip strength were collected biannually since 2006. My analysis drew on data from the five waves conducted in 2006, 2008, 2010, 2012, and 2014, in which data on all three measures were available.

The anchor year of my study was 2006. Because of a refreshment sample added to the SOEP in this year, the data are representative of the German population aged 17 years and older in 2006. From all individuals participating in the 2006 SOEP wave (*N* = 20,590), a random sample of 7,143 individuals was selected to participate in tests of grip strength. My analysis was based on this subsample of individuals. I further restricted this sample to West Germans, excluding immigrants (*n* = 841) and persons who lived in the German Democratic Republic (GDR) before reunification in 1990 (*n* = 1,900).^1^ These restrictions ensured that individuals shared a common context with regard to education degrees and life conditions. I further constrained the sample to individuals who were 25 to 75 years old at their initial observation in 2006. This restriction resulted in a cohort range of 1931 and 1981, excluding individuals born before or after these years (*n* = 754). Most men born before 1931 were enlisted to fight in World War II and might constitute a selective group of war survivors. The minimum age of 25 years at first observation ensures that the process of education attainment was completed before or during the observation period for all individuals included in the sample. Finally, I removed respondents without information on education (*n* = 13). After all restrictions, my analytic sample consisted of 3,635 individuals comprising 9,869 person-years.

### Measures of Health

Self-rated health (SRH) was assessed in the SOEP by the survey question, “How would you describe your current health?” Respondents answered on a scale from 1 (very good) to 5 (bad). I reverse-coded this variable so that lower values indicated worse health. The response rate to this question is the highest among all health measures: only 0.2 % of data are missing.

The Physical Component Scale (PCS) is a health indicator developed to assess the objective health status of respondents (Steiber 2016; Ziebarth 2010). In the SOEP data, the PCS score was calculated by the SOEP group (Andersen et al. 2007) on the basis of the SF-12v2 questionnaire (Fleishman et al. 2010). SF-12 is a widely used measurement instrument based on a multi-item scale evaluating the following aspects of physical health: (1) limitations in physical activities because of health problems; (2) limitations in usual role activities because of physical health problems; (3) bodily pain; and (4) general health perceptions (Ware and Sherbourn 1992). The PCS is standardized by the SOEP group to a mean value of 50 and a standard deviation of 10. More information on the items and on the calculation procedure can be found in the online appendix. The share of missing data on this item is low, at 1.9 %.

Grip strength in kilograms was measured using the Smedley S Dynamometer TMM Tokio 100kg (Ambrasat and Schupp 2011). Previous research has shown that values taken with a Smedley dynamometer strongly correlate with those taken with the more commonly used Jamar dynamometer (Yorke et al. 2015). Interviewers were trained to perform the test correctly. Two measures from each hand were taken. The share of missing data for grip strength is only slightly higher than for PCS, at 3.2 %.

In the analysis, I included grip strength as (1) a continuous measure of grip strength and (2) a dichotomous measure indicating whether it crossed a cutoff value identifying a “weak grip.” The additional measure of weak grip had one main advantage. Declines in grip strength may not necessarily identify relevant declines in physical health if they occur at higher levels of grip strengths. Studies have supported this consideration, showing that cutoff values of weak grip predicted mortality, disability, and functional limitations better than average levels of grip strength (Steiber 2016).

For the continuous measure, I took the average of all four measurements for individuals with at least one valid measure. The dichotomous measure defines “weak grip” for those whose grip strength was at least 1 sex-specific standard deviation below the sex-specific average. This is one of the common cutoff specifications next to 1.5 and 2 standard deviations, which are suitable for younger populations (Steiber 2016).

Table 1 shows descriptive statistics for each of the health measures separately for men and women. Average levels of SRH (3.28 vs. 3.36) and PCS (48.7 vs. 49.6) were similar for men and women. Average grip strength was much higher among men (45.75 kg) than among women (28.56 kg). The prevalence of weak grip was by definition of the measure similar in both sexes, at 14 % among men and 15 % among women.

**Table 1:** Descriptive statistics

	Men				Women			
	Mean	SD	Min.	Max.	Mean	SD	Min.	Max.
Health Measures								
Self-rated health^a^	3.36	0.88	1	5	3.28	0.90	1	5
Physical Component Scale^b^	49.6	9.46	13.1	73.8	48.7	9.96	12.9	73.1
Average grip strength (in kg)^c^	45.75	10.05	3.7	82.13	28.56	6.75	1.8	64.18
Weak grip^d^	0.14		0	1	0.15		0	1
Age and Cohort								
Age	54.1	13.5	25	83	53.8	13.5	25	83
Mean-centered age	0.013	13.5	–29.1	28.9	–0.30	13.5	–29.1	28.9
Cohort	1955.5	13.2	1931	1981	1955.7	13.2	1931	1981
Mean-centered cohort	–4.51	13.2	–30	20	–4.69	13.2	–30	20
Education^e^								
Lower	0.46		0	1	0.50		0	1
Intermediate	0.30		0	1	0.49		0	1
Higher	0.24		0	1	0.36		0	1
Panel Attrition								
Died^f^	0.039		0	1	0.021		0	1
Left^g^	0.25		0	1	0.25		0	1
Observation Period								
Number of waves	3.68	1.36	1	5	3.69	1.37	1	5
Survey year	2010	2.81	2006	2014	2010	2.81	2006	2014
Number of Individuals	1,756				1,879			
Number of Observations	4,799				5,070			

*Source:* Data are from SOEP, v.32 release 2016.

^a^ Reverse-coded so that lower values indicate worse health.

^b^ Summary index of physical health derived from SF-12 instrument.

^c^ Averaged across all measurements (up to two for each hand).

^d^ Weak grip is defined as 1 sex-specific SD below the sex-specific mean value.

^e^ Lower = CASMIN 1a–1c; intermediate = CASMIN 2a–2cvoc; and higher = CASMIN 3a–3b.

^f^ Fraction of respondents who died between 2006 and 2014.

^g^ Fraction of respondents who left the panel between 2006 and 2014 for reasons other than death.

Because these health indicators were measured on different scales, I standardized the scores of SRH, PCS, and grip strength separately by gender to allow for a direct comparison between these indicators. Because the indicator of weak grip is dichotomous, it cannot be similarly standardized. I will thus compare only the main pattern of education differences in weak grip with the main patterns shown by the other measures. Results on the original scales are shown in Fig. A1 in the online appendix.

### Measures of Age and Cohort

Age was measured in years, ranging from 25 to 83 across the observation window. I centered the age variable at the mean of 54 years (see Table 1). Birth cohort, measured as age at the anchor year in 2006, ranged between 25 and 75 corresponding to birth years from 1931 until 1981. In multivariate models, cohort was centered at the value of 55 corresponding to the birth year of 1951.^2^

The parameterizations of age and cohort effects on the health outcomes were based on two criteria: (1) similarity between observed and fitted data examined by diagnostic plots, and (2) the BIC criterion. This resulted in different functional forms of age for different health outcomes. In the models for SRH and PCS, I included only linear term of age for both men and women. In the model for men’s grip strength, I included linear and quadratic terms of age; in the model for women’s grip strength, I included linear, quadratic, and cubic terms of age. The model of weak grip included linear and quadratic terms of age for women, and linear, quadratic, cubic, and quartic terms of age for men.

### Measure of Education

**Fig. 1:**
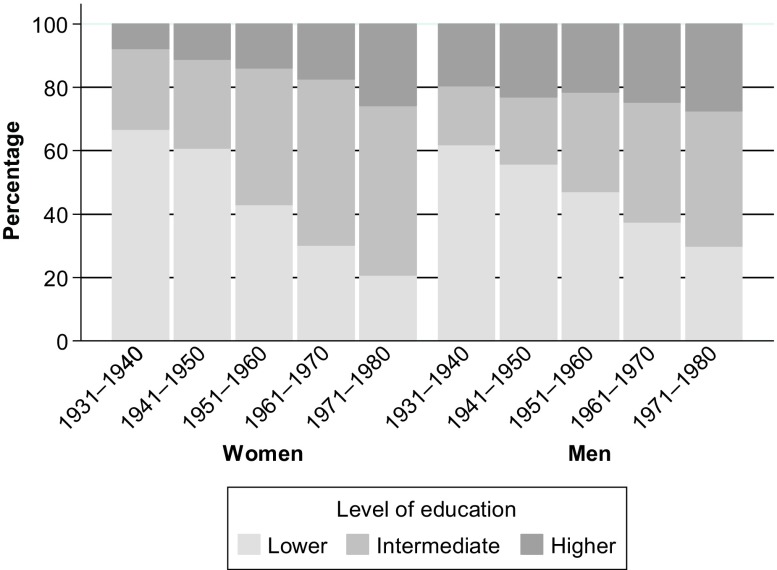
Distribution of education levels across cohorts. Lower education = CASMIN 1a–1c; intermediate education = CASMIN 2a–2cvoc; and higher education = CASMIN 3a–3b. *Source:* Data are from SOEP, v.32 release 2016.

I measured education degrees by the CASMIN classification. This variable indicates the highest education degree reported by respondents within the observation period. I grouped the CASMIN into three categories. The bottom category comprises individuals holding lower secondary degrees with completed vocational qualification or less (CASMIN 1a–1c). Intermediate education ranges from intermediate secondary degrees to higher secondary degrees with vocational qualification (CASMIN 2a–2cvoc). The top category includes respondents holding tertiary degrees (CASMIN 3a–3b). Figure 1 shows the distribution of education categories across cohorts.

### Analytic Strategy

I estimated change in SRH, PCS, and grip strength using hierarchical linear regression models (HLM) and changes in weak grip using a hierarchical linear probability model (Raudenbush and Bryk 2002). My data included up to five observations (Level 1) per person (Level 2), measured at biannual intervals. The HLM estimation accounts for heterogeneity in health trajectories, allowing individual age trajectories to differ in their starting levels (random intercepts) and rates of change (random slopes). The estimation of HLM provides information about mean health trajectories (growth curves) as well as individual variation around the average curves.

As discussed in the section on theoretical background, an appropriate analytical strategy to estimate change in the relationship between education and health is to account simultaneously for change with age, change across cohorts, and their interactions (Lynch 2003; Mirowsky and Ross 2008; Willson et al. 2007). This approach translates into an empirical model that includes age, cohort, and education as well as twofold and threefold interactions between these variables.

The growth curves for each of the health outcomes of respondent *i* at time *t* are calculated as shown in Eq. (1) for Level 1 variables and Eq. (2) for Level 2 variables^3^ (see also Willson et al. 2007):

1 $$ {Health\ measure}_{it}={\uppi}_{0i}+{\uppi}_{1i}{age}_{it}+{e}_{it}, $$

where *i* = 1, . . . , *N* are individuals in the sample; π_0*i*_ is an individual-specific intercept; and π_1*i*_ is the growth rate for individual *i*. This model estimates effects of individual characteristics on the intercepts (π_0*i*_) and slopes (π_1*i*_) of Level 1 variables.

2 $$ {\displaystyle \begin{array}{l}{\uppi}_{0i}={\upbeta}_{00}+{\upbeta}_{01}{intermediate\ education}_i+{\upbeta}_{02}{high\ education}_i+{\upbeta}_{03}{cohort}_i+\\ {}{\upbeta}_{04}{cohort}_i\times intermediate\ {education}_i+{\upbeta}_{05}c{ohort}_i\times \\ {}\begin{array}{l} high\ {education}_i+{\upbeta}_{0q}{controls}_i+{r}_{0i},\\ {}{\uppi}_{1i}={\upbeta}_{10}+{\upbeta}_{11} intermediate\ {education}_i+{\upbeta}_{12} high\ {education}_i+{\upbeta}_{13}{cohort}_i+\\ {}\begin{array}{l}\ {\upbeta}_{14}\ {cohort}_i\times intermediate\ {education}_i+{\upbeta}_{15}{cohort}_i\times \\ {}\  high\ {education}_i+{r}_{1i},\end{array}\end{array}\end{array}} $$

where β_*pq*_ are the effects of individual characteristics on intercept π_0*i*_ and slope π_1*i*_, and *r*_*pi*_ is an error term for unmeasured time-constant characteristics of individual *i*.

Combining Eqs. (1) and (2) yields the following equation for the models shown in Table A1 in the online appendix:

3 $$ {Health\ measure}_{it}=\left[{\upbeta}_{00}+{\upbeta}_{10}{age}_{it}+{\upbeta}_{01} intermediate\ {education}_i+{\upbeta}_{02} high\ {education}_i+{\upbeta}_{03}{cohort}_i+{\upbeta}_{04}{cohort}_i\times intermediate\ {education}_i+{\upbeta}_{05}{cohort}_i\times high\ {education}_i+{\upbeta}_{0q}{controls}_i+{\upbeta}_{11}{age}_{it}\times intermediate\ {education}_i+{\upbeta}_{12}{age}_{it}\times high\ {education}_i+{\upbeta}_{13}{age}_{it}\times {cohort}_i+{\upbeta}_{14}{age}_{it}\times intermediate\ {education}_i\times {cohort}_i+{\upbeta}_{15}{age}_{it}\times high\ {education}_i\times {cohort}_i\right]+\left[{e}_{it}+{r}_{0i}+{r}_{1i}{age}_{it}\right]. $$

Given the theoretical considerations and previous findings about gender differences, I performed all analyses separately for men and women.

An issue that needs to be addressed in analyses of change in life course research on health inequality is selection related to health. To assess the role of selective attrition from the panel, I examined (1) the dropout risk among lower- and higher-educated people, (2) initial health levels among lower- and higher-educated dropouts compared with those who stayed in the panel, and (3) applied inverse probability weighting to correct for potential bias due to attrition. Descriptive results (Table A2 in the online appendix) show that lower-educated men and women were more likely to die than higher-educated men and women. In line with previous evidence (Baeten et al. 2013), this suggests that selective mortality led to positive health selection among lower-educated individuals who remained under observation.

Further, the results in Table A2 in the online appendix show that dropout for reasons other than death was barely related to health levels at initial observation. Although these findings provide no reasons to expect bias due to panel attrition, it is still possible that *changes* in health were related to different types of attrition.

To correct for this potential source of bias, I used inverse probability weighting (IPW) (Wooldridge 2002). The variables included in the models to calculate inverse probability weights were each of the four health measures, age, education, and interactions between each of these variables measured at *t* – 1. I calculated the weights separately for men and women. The results of the main analysis remained almost unchanged after I used IPW: the predictor variables were only weakly associated with the probability of participating in each of the waves (estimates not shown).

The results from the weighted HLM models are shown in Table A1 in the online appendix. In the presence of twofold and threefold interaction effects, the coefficients in Table A1 cannot be interpreted straightforwardly: they show estimates for only one specific combination of values. For example, the main effect of cohort pertains to lower-educated people at age 54, and the interaction effects between education and age pertain to the value at which the cohort was centered (to those born in 1951).

**Fig. 2:**
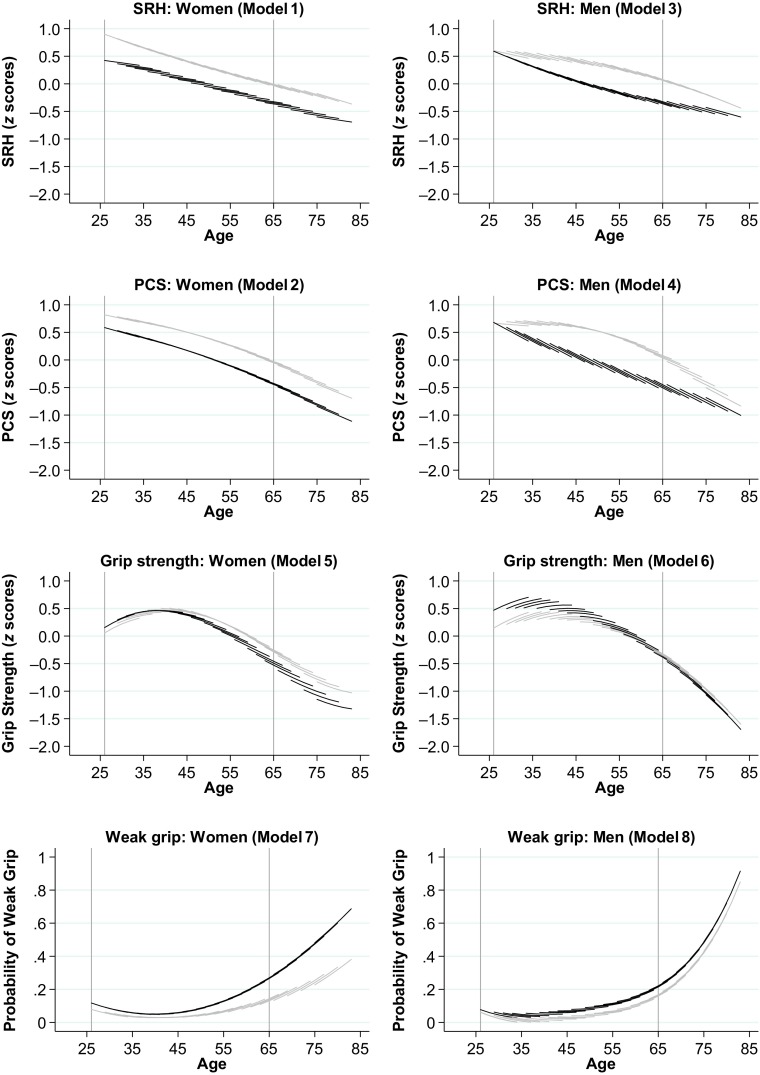
Predicted aging vectors of self-reported and objective health measures. Predictions are based on Models 1–8 in Table A1 of the online appendix. Black lines = lower education. Gray lines = higher education. *Source:* Data are from SOEP, v.32 release 2016.

To facilitate the interpretation, I present age-vector graphs (Mirowsky and Kim 2007) that show age-related changes in average health levels for different birth cohorts of lower- and higher-educated people (Fig. 2). In addition, Table 2 presents the corresponding marginal effects for education differences and their confidence intervals for each measure of health, changes in these differences with age, and tests of differences between *z*-standardized measures of SRH and PCS (SRH – PCS) as well as between *z*-standardized measures of SRH and grip strength (SRH – grip strength).

**Table 2:** Marginal education differences at age 26 and age 65, by health measure and gender

	SRH		PCS		Grip Strength		Weak Grip	
	Men,	Women,	Men,	Women,	Men,	Women,	Men,	Women,
	Model 1	Model 2	Model 3	Model 4	Model 5	Model 6	Model 7	Model 8
Education Differences								
At age 26	0.011	0.476	–0.014	0.231	–0.322	–0.097	–0.016	–0.039
	[–0.365, 0.388]	[0.074, 0.878]	[–0.317, 0.289]	[–0.097, 0.560]	[–0.689, 0.044]	[–0.458, 0.264]	[–0.092, 0.059]	[–0.153, 0.075]
At age 65	0.391	0.387	0.437	0.391	0.038	0.259	–0.053	–0.151
	[0.202, 0.581]	[0.139, 0.634]	[0.261, 0.614]	[0.148, 0.634]	[–0.114, 0.191]	[0.077, 0.442]	[–0.125, 0.018]	[–0.231, –0.066]
Change from age 26 to 65	0.380	–0.089	0.451	0.160	–0.360	0.356	–0.037	–0.112
	[0.193, 0.567]	[–0.244, 0.065]	[0.325, 0.578]	[0.074, 0.245]	[–0.575, –0.147]	[0.178, 0.535]	[–0.217, –0.041]	[–0.141, –0.078,]
Number of Observations	4,608	4,824	4,608	4,824	4,608	4,824	4,608	4,824

*Notes:* Average marginal differences are shown. Education differences in SRH, PCS, and grip strength are expressed in standard deviations. Differences in weak grip are expressed in percentage points. Values in brackets are 95 % confidence intervals. Estimates are based on Models 1–8 in Table A1 of the online appendix. Differences at ages 26 and 65 are predicted mean differences between higher- and lower-educated respondents. Positive values of SRH, PCS, and grip strength as well as negative values of weak grip indicate better health among higher-educated than lower-educated respondents. Cohort is centered at ages 26 and 65. Change with age is calculated as a difference between the predicted mean differences at age 26 and the predicted mean differences at age 65.

*Source:* Data are from SOEP, v.32 release 2016.

## Results

Figure 2 shows model-based predictions for change in all health measures. Health trajectories of higher-educated people are represented by gray curves, and health trajectories of lower-educated people are represented by black curves. In the plots for SRH, PCS, and grip strength, lower values indicate worse health; in the plots for weak grip, higher values indicate worse health. The *y*-axes of the figures are defined for the predicted average values of the four outcomes at fixed values of age, cohort, and education. To allow for cohort effects, I fixed the variable for cohort at 20 values of age at initial observation, counting in three-year intervals from the age of 26 (i.e., birth year of 1980) to the age of 75 (i.e., birth year of 1931). As a result, the plots show cohort-specific curves, whereby the length of each curve indicates each cohort’s age at the beginning and at the end of their observation period. Cohort effects are visible at the age overlaps across cohorts. If cohort effects are small, the cohort-specific curves connect; as cohort effects increase, the pattern appears ragged (Mirowsky and Kim 2007).

The results presented in Fig. 2 provide answers to the key question of the present study: namely, whether and to what extent life-course trajectories of health—and education differences therein—depend on the health outcome. To evaluate age patterns and effect sizes, I compared education differences at the initial age of 26 with education differences at the age of 65, indicated by reference lines in each plot. This age range covers an important part of the adult life course during which education differences in health unfold. Moreover, before the age of 65, study cohorts of lower- and higher-educated people are still largely unaffected by selective mortality.

To assess variability in the estimates, Table 2 shows the corresponding marginal effects for education differences along with their confidence intervals for SRH, PCS, and grip strength at the initial age of 26 and at the age of 65 as well as change in education differences in these health measures across this age interval.

Two central findings emerged from the analysis. First, as shown in Fig. 2 and Table 2, the pattern of health inequality unfolding over the life course looks similar for the measures of SRH and PCS. For men, education differences on both measures increased with age, and these changes were substantial in magnitude (columns 1 and 3, Table 2). There were no education differences in men’s SRH and PCS at the initial age. Until the age of 65, the gap on both measures grew to approximately 40 % of a standard deviation. Moreover, the cohort pattern among men suggests that the life course increase of education differences was less pronounced among older cohorts and intensified in newer cohorts of men (Fig. 2). However, these cohort effects were small and not statistically significant.^4^ Among women, initial education differences (age 26) in SRH were large and remained stable across the following four decades of life (until age 65). Initial differences in women’s PCS amounted to one-quarter of a standard deviation. Until the age of 65, these differences increased by 16 % of a standard deviation.

Second, men’s and women’s health trajectories of the exact same samples look entirely different if measured by the objective measures of average grip strength and the probability of weak grip. Among women, education differences in grip strength increased substantially with age. At the age of 26, lower-educated women had a slightly stronger grip than higher-educated women (column 5, Table 2). These differences reversed after the age of 40, with higher-educated women showing slower declines in average grip strength. By age 65, grip strength of higher-educated women exceeded grip strength of lower-educated women by approximately one-third of a standard deviation (or 2 kg). The cohort trend among women suggests larger differences in older cohorts, but these cohort effects were small.

The measure of weak grip confirmed these results for women. At age 26, the probability of weak grip was estimated at approximately 10 % both among lower- and higher-educated women (Fig. 2). By age 65, the probability of weak grip increased to approximately 15 % for higher-educated women, whereas it almost tripled to approximately 30 % for lower-educated women. Until the age of 80, these education differences grew further, increasing to about 30 percentage points.

Among men, a contrasting pattern emerged. At the initial age, lower-educated men had considerably higher grip strength, surpassing their higher-educated counterparts by one-third of a standard deviation (3 kg). These differences converged with age. At age 65, no education differences were found in men’s grip strength. Only at ages older than 65, higher-educated men were slightly stronger than lower-educated men.

In the probability of men’s weak grip, no differences were found at the initial age of 26. Around age 40, an advantage of higher-educated men emerged. This gap increased only slightly (by roughly 4 percentage points) across middle and later stages of the life course (Table 2, column 7).

### Additional Analyses

In the additional analyses, I examined the unexpected results for men’s grip strength in more detail, addressing two post hoc explanations.

The first possible explanation is differences in body weight. As research has shown, higher body weight is associated with stronger grip for men but not for women (Gale et al. 2007). Given that lower-educated men are heavier, on average, than higher-educated men, this difference may be reflected in higher grip strength among lower-educated men. The second potential explanation concerns differences in occupations. Lower-educated men more often work in manual occupations, and manual work could contribute to muscle mass and strength. Importantly, both of these factors—higher body weight and manual work—constitute substantial health risks in the longer run (Kjellsson 2013), although they may contribute to higher grip strength in younger adulthood (Kröger et al. 2016).

Figure A2 in the online appendix illustrates the results obtained from the additional analyses. Once body weight was controlled for (middle plot), lower-educated men’s early-life advantage in grip strength was reduced. In later life, instead, the gap between higher- and lower-educated men increased slightly, with grip strength declining faster among the lower-educated. The additional control for manual occupation (right-hand plot) entirely explained the advantage of lower-educated men in terms of grip strength in younger adulthood as well as their small disadvantages in later life.

## Discussion

The cumulative (dis)advantage hypothesis predicts that education differences in health increase with age (Ross and Wu 1996). Although many studies have supported this prediction, the evidence is limited in an important way. All previous tests were based on self-reported health measures. Despite the numerous and widely recognized benefits of these measures, recent research has raised concerns about their validity in terms of key analytical constructs in studies of the cumulative (dis)advantage hypothesis, including education differences, age differences, cohort differences, and gender differences. In view of potential bias related to these issues, it remained unclear whether and to what extent previous conclusions about health inequality over the life course were affected by group differences in the validity of self-reported health measures.

To my knowledge, the present study is the first to address this issue by offering a systematic comparison on self-reported and objective health outcomes measured on the same samples observed across a period of eight years and covering the full age range of the adult life course. The outcomes explored here are indicators recognized in the literature as valid and reliable measures of general physical health, including self-rated health, the Physical Component Scale, and objective measures of grip strength and weak grip.

The main question guiding my analysis was whether education differences in health take similar shapes if assessed by self-reported and objective measures of physical health. The answer is no. For both men and women, conclusions regarding the main pattern predicted by the cumulative (dis)advantage hypothesis were reversed depending on whether the analysis was based on self-reported or objective measures. When health was measured by self-reported measures, the prediction of cumulative (dis)advantage hypothesis was consistent with men’s, but not women’s, health trajectories. If health was measured by grip strength and the probability of weak grip, the prediction of the cumulative (dis)advantage hypothesis was consistent with women’s, but not men’s, health trajectories.

Although the results for women showed different patterns for self-reported and objective measures, the discrepancy was not as large as for men. The main difference for women concerned early adulthood. At younger ages, education gaps were large in women’s self-rated measures but absent in women’s objective measures. A possible explanation is that self-rated measures of health may capture risky health behaviors that did not yet turn into actual health problems detectable with objective health measures (Peersman et al. 2012; Zajacova et al. 2017). In a Swedish study, men but not women considered risky health behaviors in their self-reports of general health even if the negative health effects of these behaviors had not transpired yet (Peersman et al. 2012). In a study from the United States, no gender differences in the effect of health behaviors on self-rated health were found (Zajacova et al. 2017). In the absence of similar studies for the German context, it is unclear whether health behaviors are more important for women’s than for men’s self-reports of health.

Among men, the differences between self-reported and objective measures of health were much larger than among women. Self-reported measures showed substantial advantages of higher-educated men, which increased with age. Objective measures showed advantages of lower-educated men at younger ages and hardly any education differences in later life. The findings for self-reported measures are consistent with the pattern reported for German men in a recent study of the cumulative (dis)advantage hypothesis (Leopold and Leopold 2018). Moreover, other studies have found substantial education differences among German men in chronic conditions and functional limitations (Lampert et al. 2017). This evidence, albeit cross-sectional, contradicts the findings obtained with grip strength as an objective measure of men’s physical health, which showed almost no differences between higher- and lower-educated men in middle and later stages of the adult life course.

My results on men’s grip strength are consistent with previous cross-sectional evidence, which showed much smaller education gaps in men’s than in women’s grip strength (Botoseneanu et al. 2015; de Lima et al. 2017; Strand et al. 2016). These studies have speculated that differences in grip strength among men might be suppressed because of manual work and excess body weight of lower-educated men—two health risks that are associated with higher levels of grip strength. I was able to examine these factors in a longitudinal design and found that both were indeed influential, but mainly for explaining the advantages of lower-educated men from early until middle stages of the adult life course. These factors did not explain why the measure of grip strength indicated no major health differences between higher- and lower-educated men, contradicting extant evidence showing large differences in terms of chronic conditions and functional limitations (Lampert et al. 2017).

The question that arises from these findings is whether the analyses of an objective measure of health provides more or less accurate evidence on the cumulative (dis)advantage hypothesis than analyses of self-reported measures of health. This question touches on the even more general question of which measure is more suitable for assessing differences in general physical health over the life course.

Although current knowledge is too limited to provide a definitive answer to these questions, it is important to consider the criteria on which different measures are evaluated. First, regarding reporting heterogeneity bias, objective measures of grip strength and weak grip are clearly superior. As health measures assessed by a dynamometer, grip strength overcomes important limitations of self-reported measures, which rely on an assumption that people in different groups apply similar criteria when evaluating their health status. As several studies have shown, this assumption is unwarranted in studies of health differences over the life course between education groups, genders, and cohorts (Dowd and Zajacova 2010; Peersman et al. 2012; Schnittker and Bacak 2014; Zajacova and Woo 2016). An accurate representation of all these factors has been shown to be essential in studies of health inequality over the life course.

Second, when accuracy is evaluated on the basis of covering a broad range of health problems, the answer is less clear, and it may differ between men and women. One of the main reasons why some health measures are considered broader and more general than others is the strength of their correlation with all-cause mortality. If mortality is a valid criterion for accuracy, results that fit better with education differences in mortality can be considered more accurate. As a recent demographic study from Germany showed, education differences in life expectancy are much larger among men than among women (Luy et al. 2015). In this sense, self-reported measures may be more accurate than grip strength despite reporting bias.

This conclusion is still preliminary. More research is needed to understand the extent to which declines in grip strength reflect common health risks that produce education differences in other measures of general physical health and mortality for men and women. Future research needs to assess not only the strength of the correlations between SRH, PCS, and grip strength and mortality as well as other objectively measured health risks, but also age-related changes in these correlations as well as differences by education and gender. However, in light of the findings of the present study, using grip strength as the main or only measure of general physical health appears to be an insufficient alternative to self-reported measures of physical health, despite the important analytical benefits associated with grip strength.

Looking at the big picture of research on health inequality, the current study makes two main contributions. First, it demonstrates that the choice of health measure plays a crucial role in tests of the cumulative (dis)advantage hypothesis—one of the most prominent sociological hypotheses on health inequality. Basic conclusions may be reversed when assessing different measures of general physical health—all of which are widely recognized and frequently used in research—on the exact same samples of respondents. The contribution of the current study is to uncover these differences, establishing the central role of health outcomes in the study of health inequality over the life course.

Second, the present study contributes to a broad literature in the fields of demography, economics, epidemiology, and medical sciences that focuses on the question of how general health in a population can and should be measured. My results show that the measure of grip strength—one of the most commonly used objective measures of health in representative surveys—was not sensitive to education differences for men. Based on the measure of grip strength alone, the evidence would suggest that men’s health trajectories over the life course do not differ by education, at least not in major ways. This finding contrasts with a large body of evidence on education gradients in men’s health, raising concerns about the measure of grip strength as an indicator of general physical health for men.

## Electronic supplementary material

ESM 1 (PDF 542 kb)

## Footnotes

1 Most respondents in this sample received their education degrees before reunification. Education degrees of the German Democratic Republic and their implications for health across the life course are hardly comparable with West German degrees. Thus, East Germans could be considered only for a separate analysis. However, the number of cases was not sufficient to meet the analytical requirements of this study. Because data about grip strength were collected only in a comparatively small random sample of SOEP participants, the East German sample for which data were available consisted of only 740 men and 837 women. When further broken down by gender, age, and cohort, case numbers within analytic cells became too small for a robust analysis.

2 The observation window of eight years provided only limited age overlaps between cohorts. Therefore, cohort effect could not be examined in detail.

3 The equation pertains to Models 1–4, in which only the linear term of age was used.

4 I evaluated the statistical significance of cohort effects on each health measure by calculating marginal education differences and their confidence intervals for different values of cohort and age (results not shown). None of the differences between cohorts were statistically significant.
