# Supplementary material for: Health Measurement and Health Inequality Over the Life Course: A Comparison of Self-rated Health, SF-12, and Grip Strength
Source: Demography. 2019 Mar 5;56(2):763–84. doi: 10.1007/s13524-019-00761-x (PMC6449289; doi:10.1007/s13524-019-00761-x)
Supplement: Supplementary file 1 — (PDF 542 kb) [file 13524_2019_761_MOESM1_ESM.pdf]

Online Appendix

The calculation of the Physical Component Scale

In order to calculate a summary index of physical health, the SOEP group first computed four separate scales of physical health: 1) The bodily pain scale, which is based on the following item: "Please think about the last four weeks. How often did it occur within this period of time that you had strong physical pains always, often, sometimes, almost never, or never?" 2) Physical functioning, which was assessed by the following questions: "When you ascend stairs, i.e., go up several floors on foot: Does your state of health affect you greatly, slightly or not at all?"; "And what about having to cope with other tiring everyday tasks, i.e., when one has to lift something heavy or when one requires agility: Does your state of health affect you greatly, slightly or not at all?" 3) The role physical scale, which was computed from the following items: "Please think about the last four weeks. How often did it occur within this period of time that due to physical health problems you achieved less than you wanted to at work or in everyday tasks; that due to physical health problems you were limited in some form at work or in everyday tasks always, often, sometimes, almost never, or never?" 4) For general health, the same question as for self-rated health was used. To compute the PCS, the SOEP group first calculated "norm-based scoring" using all respondents who had valid answers on each of the items from the wave of 2004. Each of the four sub-scales was rescaled to a range of 0 to 100 and z-standardized. Next, the scales were linearly transformed to a mean value of 50 and a standard deviation of 10. Finally, factor analysis have been conducted, which showed that all items strongly loaded on one dimension (Andersen et al 2007:178). A summary score was computed from the z-standardized sub-scales. The final Physical Component Scale (PCS) has been standardized in the same way.

TABLE A1: HIERARCHICAL LINEAR MODELS

	M1<br>SRH<br>Women	M2<br>PCS<br>Women	M3<br>SRH<br>Men	M4<br>PCS<br>Men	M5<br>GS<br>Women	M6<br>Weak grip<br>Women	M7<br>GS<br>Men	M8<br>Weak grip<br>Men
Intercept	-0.16**	-0.090	-0.11*	-0.11*	-0.029	0.18***	-0.14	0.19***
	(0.05)	(0.05)	(0.05)	(0.05)	(0.08)	(0.03)	(0.08)	(0.03)
Age (centered at age 54)	-0.011	-0.029***	-0.026***	-0.041***	0.19**	-0.026	0.12***	-0.10
	(0.01)	(0.01)	(0.01)	(0.01)	(0.07)	(0.02)	(0.04)	(0.08)
Cohort (centered at 1951)	-0.0096	-0.0023	0.0071	0.014	-0.011	0.00081	-0.016**	0.0022
	(0.01)	(0.01)	(0.01)	(0.01)	(0.01)	(0.00)	(0.01)	(0.00)
Age * Cohort	-0.000016	-0.00024	0.00022	0.000042	-0.00062	0.000026	0.00042	-0.000055
	(0.00)	(0.00)	(0.00)	(0.00)	(0.00)	(0.00)	(0.00)	(0.00)
Education (ref. Lower)								
Intermediate	0.22**	0.18*	0.11	0.18*	0.096	-0.016	-0.034	-0.042
	(0.08)	(0.07)	(0.08)	(0.08)	(0.07)	(0.02)	(0.08)	(0.03)
Higher	0.39**	0.36***	0.44***	0.53***	0.19	-0.070*	-0.030	-0.045
	(0.12)	(0.10)	(0.10)	(0.09)	(0.10)	(0.03)	(0.07)	(0.03)
Education * Age								
Intermediate * Age	-0.0080	0.0023	0.0063	0.014	-0.0073	0.00026	0.0099	0.0023
	(0.01)	(0.01)	(0.01)	(0.01)	(0.01)	(0.00)	(0.01)	(0.00)
Higher * Age	-0.014	0.0033	0.00026	0.014	-0.0020	-0.0032	0.0032	-0.0010
	(0.01)	(0.01)	(0.01)	(0.01)	(0.01)	(0.00)	(0.01)	(0.00)
Education * Cohort								
Intermediate * Cohort	0.0092	-0.0037	-0.0083	-0.017	0.0038	0.0013	-0.0064	-0.0026
	(0.01)	(0.01)	(0.01)	(0.01)	(0.01)	(0.00)	(0.01)	(0.00)
Higher * Cohort	0.014	-0.00030	0.00035	-0.016	0.0099	-0.0025	0.0043	0.00023
	(0.01)	(0.01)	(0.01)	(0.01)	(0.01)	(0.00)	(0.01)	(0.00)
Education * Cohort * Age								
Intermed. * Cohort * Age	-0.000044	-0.00025	-0.00034	-0.00047	-0.000032	0.000038	-0.000033	0.000018
	(0.00)	(0.00)	(0.00)	(0.00)	(0.00)	(0.00)	(0.00)	(0.00)
Higher * Cohort * Age	0.00012	-0.000061	-0.00051	-0.00076**	-0.000067	-0.00016	-0.000095	0.0000096
	(0.00)	(0.00)	(0.00)	(0.00)	(0.00)	(0.00)	(0.00)	(0.00)
Age squared					-0.0039***	0.00032*	-0.0013***	0.0032
					(0.00)	(0.00)	(0.00)	(0.00)
Age cubic					0.000022***			-0.000046
					(0.00)			(0.00)
Age quartic								0.0000003
								(0.00)
Numberofobservations	4,824	4,824	4,608	4,608	4,824	4,824	4,608	4,608

Note: Data are from SOEP, v. 32, release 2016. Standard errors in parentheses. ** $p < 0.01$, * $p < 0.05$.

TABLE A2: Attrition Analysis

	Men						Women					
	Lower educated			Higher educated			Lower educated			Higher educated		
	Died	Left	Stayed	Died	Left	Stayed	Died	Left	Stayed	Died	Left	Stayed
% of initial sample	6.59	34.33	58.58	2.84	32.62	63.12	4.33	35.64	59.53	1.02	30.27	67.01
Average numberofwaves	3.06	2.63	4.15	3.18	2.38	4.06	2.86	2.54	4.23	2.20	2.31	4.03
Self-rated health at Wave 1	2.83	3.28	3.34	3.00	3.70	3.56	2.26	3.05	3.16	2.67	3.69	3.52
PCS at Wave 1	43.3	49.0	48.9	39.0	54.2	52.7	38.5	46.6	46.8	41.3	53.0	52.6
Grip strength at Wave 1	36.7	46.5	46.2	36.1	46.2	45.0	23.4	27.3	27.8	22	29.1	30.1
Weak grip at Wave 1	0.44	0.12	0.14	0.67	0.071	0.12	0.37	0.16	0.17	0.33	0.042	0.067
Numberofindividuals	53	276	471	12	138	267	35	288	481	3	89	197
Numberofobservations	128	597	1591	33	266	872	83	592	1,655	5	168	643

Note: Data are from SOEP, v. 32, release 2016. Stayed = Observed until last panel wave. Left = Left panel before last wave but did not die. Died = Died before the last panel wave.

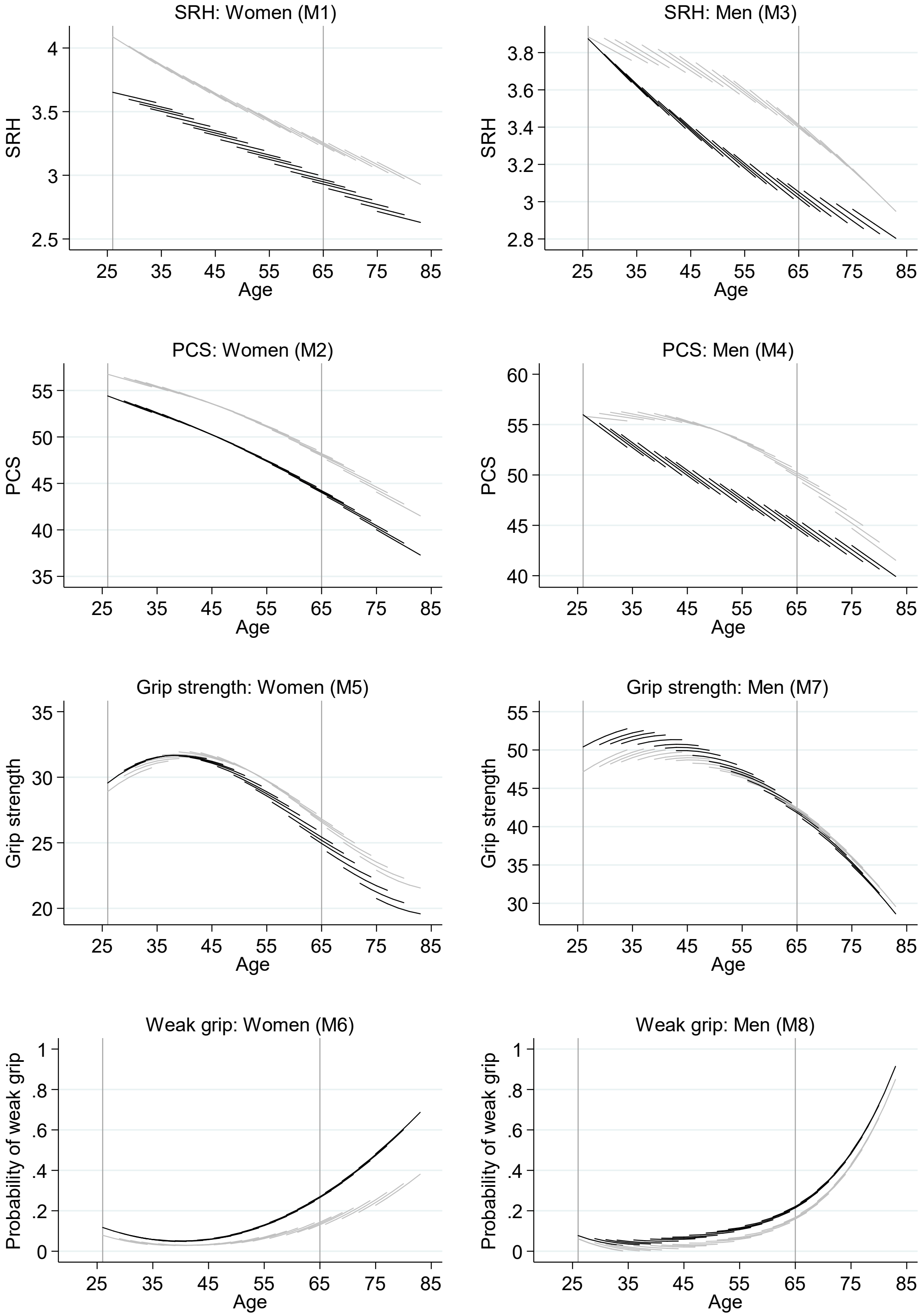

FIGURE A1. PREDICTED AGING VECTORS OF SELF-REPORTED AND OBJECTIVE HEALTH MEASURES ON ORIGINAL SCALES

Note: Data are from SOEP, v.32 release 2016. Predictions are based on models M1 – M8 from Table A1. Black lines = Lower education. Grey lines = Higher education.

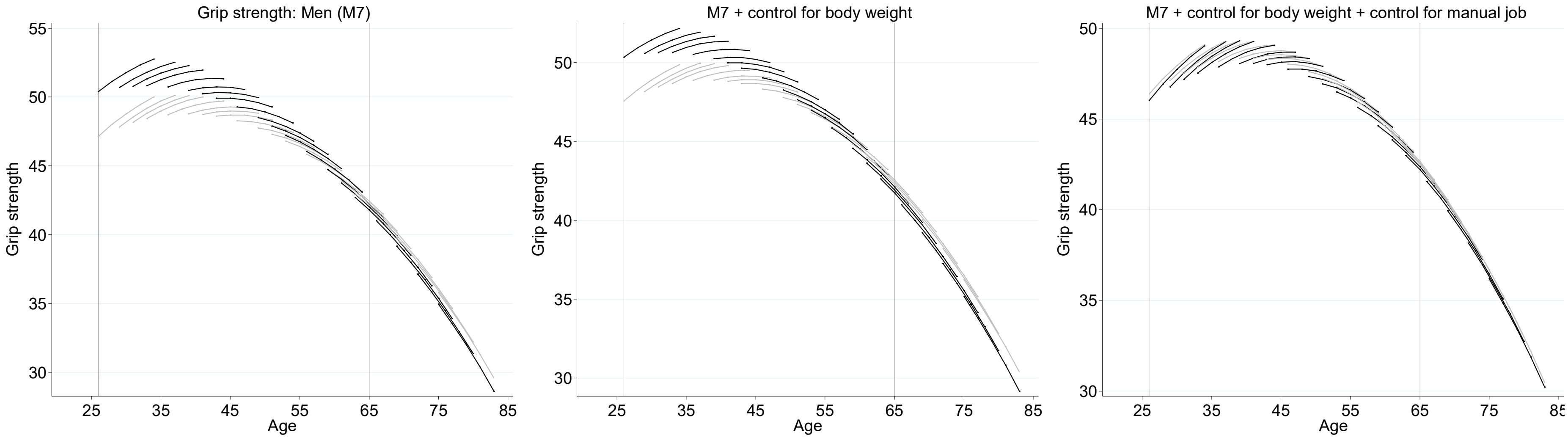

FIGURE A2. PREDICTED AGING VECTORS OF MEN'S GRIP STRENGTH

Note: Data are from SOEP, v. 32, release 2016. Predictions are based on models M7, Table A1 (left-hand panel), on model M7 with additional controls for body weight (middle panel), and on model M7 with additional controls for body weight and manual occupation (right-hand panel).
Black lines = Lower education. Grey lines = Higher education.
